# Virome of post-weaned diarrhoeic pigs and healthy cohorts in England

**DOI:** 10.1186/s12985-026-03152-y

**Published:** 2026-04-04

**Authors:** Akbar Dastjerdi, Hannah Davies, Manal Abu Oun, Indre Navickaite, Siva Karuna, Mandy Nevel, Arianna Comin, Susanna Williamson

**Affiliations:** 1 Animal and Plant Health Agency (APHA)-Weybridge, Addlestone, KT15 3NB Surrey UK; 2 Agriculture and Horticulture Development Board, Middlemarch Business Park, Siskin Parkway East, Coventry, CV3 4PE UK; 3 Department of Epidemiology, Surveillance and Risk Assessment, Swedish Veterinary Agency (SVA), Uppsala, Sweden; 4 APHA-Bury St. Edmunds, Rougham Hill, Bury St Edmunds, Suffolk, IP33 2RX UK

**Keywords:** Post weaning diarrhoea, PWD, Virome, Pigs, Metagenomics

## Abstract

**Background:**

Post-weaning diarrhoea (PWD) is a disease syndrome that negatively impacts pig health, welfare and productivity. PWD typically occurs within two weeks of weaning and coincides with significant physiological changes, including villus atrophy and increased crypt depth in the gastrointestinal (GI) tract. The GI microbiome of healthy pigs is a complex ecosystem of commensal microorganisms. Disruption of the natural integrity of the GI tract has been associated with increased colonization by both viral and bacterial pathogens.

**Methods:**

In this study, metagenomic sequencing was used to assess the presence, load, and diversity of viruses in the GI tracts of PWD-affected pigs and age-matched healthy (AMH) cohorts on commercial pig farms in England. In addition, the viromes of archived faecal samples from post-weaned pigs between four and six weeks of age, collected from diagnosis-not-reached (DNR) and diagnosis-reached (DR) enteric cases were investigated through sequencing.

**Results:**

Viruses belonging to at least ten virus families were identified in both PWD and AMH pigs including astrovirus, enterovirus, kobuvirus, smacovirus, picobirnavirus, sapovirus, parvovirus, posavirus, teschovirus, sapelovirus, rotavirus, torovirus, anellovirus and adenovirus. Co-infection with four viruses, astrovirus, enterovirus, kobuvirus and smacovirus was detected in all samples from PWD and AMH pigs. No sequence reads matching porcine coronaviruses, porcine reproductive and respiratory disease virus, porcine circoviruses, swine influenza virus, atypical porcine pestivirus or porcine teschovirus-1 were detected in either PWD or AMH faecal samples. Metagenomic analysis also identified several viruses with a higher virus load in PWD cases (astro, entero, sapelo, sapo, posa, adeno and toro-viruses), but the differences from those in AMH cases were not statistically significant. No viruses were detected in samples from archived DNR and DR cases that were not found in the PWD and AMH pigs.

**Conclusions:**

This study revealed the complexity of the virus element in the enteric microbiome in the post-weaned pigs. The role of the viruses detected and their interplay with the host and other bacterial or viral flora in inducing PWD, however, remains unclear and warrants further studies.

**Supplementary Information:**

The online version contains supplementary material available at 10.1186/s12985-026-03152-y.

## Introduction

Post-weaning diarrhoea (PWD) is a disease syndrome that adversely affects pig health, welfare and productivity. It is characterised by diarrhoea and weight loss and may result in mortality or impaired growth in surviving pigs. PWD typically occurs within two weeks of weaning, a period marked by the decline of maternal antibodies, a dietary transition from milk to less digestible solid food, and environmental stressors such as relocation and increased group size. During this time, the pig’s gastrointestinal (GI) tract undergoes significant physiological changes, including villus atrophy and increased crypt depth [1–3]. Additionally, the jejunum and colon of weaned pigs exhibit heightened secretory activity and intestinal permeability [4], which can facilitate the translocation of toxins, bacteria, or dietary antigens across the epithelial barrier. Alterations and up regulation of pro-inflammatory cytokines during the weaning process also influence intestinal integrity and epithelial function [5]. These changes collectively impair nutrient digestion and the absorptive capacity of the small intestine and may result in imbalance of the normal gut microbiota [6, 7]. Further contributing to this dysfunction is the loss of protective components found in sow’s milk, such as immune cells, lactoferrin, lysozyme and probiotic lactic acid bacteria, which ultimately reduces nutrient digestion and absorption [6–8].

The healthy pig microbiome is a complex ecosystem of commensal microorganisms, which has been shown to vary in diversity with age and across different sections of the GI tract [9]. A breakdown in the normal integrity of the GI tract has been reported to be associated with increased colonisation by viral and bacterial pathogens, including rotavirus, enteropathogenic *Escherichia coli* and *Salmonella* leading to the development of PWD [10, 11]. In addition, the ban on therapeutic use of zinc oxide in pig diets, previously used to control PWD, in the European Union in 2022 (http://ec.europa.eu/health/documents/community-register/2017/20170626136754/dec_136754_en.pdf.) may influence the incidence and duration of PWD. This regulatory change could lead to increased reliance on antimicrobials, underscoring the need for a comprehensive study of the microbiome in PWD incidents. It is essential, therefore, to detect and characterise the full spectrum of pathogens in PWD cases, as prior or co-infection with multiple pathogens may contribute to disease development. Viral metagenomics employing unbiased next-generation sequencing, is an ideal technology for virome identification. The technique enhances our understanding of virosphere diversity and provides valuable insights into the aetiology of complex disease syndromes.

This study employed metagenomic sequencing to assess the presence, load, and diversity of viruses in the GI tracts of PWD-affected pigs and age-matched healthy (AMH) cohorts on commercial pig premises in England. The findings in PWD pigs were compared with those in AMH cohort pigs to explore their associations with the enteric disease. The study also investigated several diagnosis-not-reached (DNR) and diagnosis-reached (DR) enteric cases in pigs through metagenomic identification of viral pathogens.

## Materials and methods

### Faecal samples from PWD and AMH pigs

Veterinary surgeons sampled pigs on eight farms in England according to pre-agreed criteria. Eligible pigs were required to be weaned and aged between four to six weeks at the time of sampling, and to be part of a group experiencing a PWD outbreak affecting at least 5% of animals. On each farm, faecal samples were collected from two PWD and two AMH pigs in the same cohort. PWD cases had to be diarrhoeic pigs typical of the outbreak, sampled early in the course of disease, and preferably untreated. If treatment had been given, both diseased and healthy sampled pigs had to have received the same treatment. Pigs that had been moved to hospital pens were not sampled. Faecal consistencies were scored according to agreed guidance (https://assets.elanco.com/0cec44ed-3eaa-0009-2029-666567e7e4de/f007ddb4-259b-4e91-8d47-58c36114c1fc/post-weaning-diarrhoea-pwd-faecal-score-card.pdf). PWD cases had to have diarrhoea with scores of 2 (mild), 3 (moderate) or 4 (severe), while AMH pigs had to have a score of 0 (Table 1). Faecal samples were collected from each pig in a manner that avoided environmental contamination and minimised contact with the pigs’ skin. Samples were transported to the laboratory overnight and stored at -80 °C if not processed on the day.

**Table 1:** Information on the samples used in this study

Submission ID	Sample ID	Clinical status	Rotavirus	PEDV/TGEV	Faecal score and colour
0117	0244	PWD	Neg	Neg	3, dark grey
	0245		Neg	Neg	3, dark grey
	0246	AMH	Neg	Neg	0, dark grey
	0247		Neg	Neg	0, dark grey
0099	9734	PWD	Neg	Neg	4, dark grey
	9735		Neg	Neg	4, dark grey
	9736	AMH	Neg	Neg	0
	9737		Rotavirus, not A-D	Neg	0
0082	9572	PWD	Neg	Neg	2, greyish
	9573		Neg	Neg	1, greyish^a^
	9574	AMH	Neg	Neg	0, greyish
	9575		Neg	Neg	0, greyish
0083	9583	PWD	Neg	Neg	4, mustard orange
	9584		Neg	Neg	3, light brown
	9585	AMH	Neg	Neg	0, dark grey
	9586		Neg	Neg	0, dark grey
0086	9641	PWD	Neg	Neg	4, greenish
	9642		Neg	Neg	4, yellowish
	9643	AMH	Neg	Neg	0, greyish
	9644		Neg	Neg	0, greyish
0054	2307	PWD	Neg	Neg	3, grey
	2308		Rotavirus C	Neg	3, grey
	2309	AMH	Neg	Neg	0, grey
	2310		Neg	Neg	0, grey
0055	5054	PWD	Neg	Neg	4, yellowish
	5055		Rotavirus, not A-D	Neg	4, greyish
	5056	AMH	Rotavirus, not A-D	Neg	0, greyish
	5057		Rotavirus, not A- D	Neg	0, greyish
0061	3044	PWD	Neg	Neg	4, green-yellow
	3045		Neg	Neg	4, green-yellow
	3046	AMH	Rotavirus, not A-D	Neg	0, greyish
	3047		Neg	Neg	0, greyish
0071	3338	PWD	Neg	Neg	3, grey
	3339		Neg	Neg	4, grey
	3340	AMH	Neg	Neg	0, grey
	3341		Rotavirus, not A-D	Neg	0, grey

The table also includes viral laboratory results, and faecal scores for each faecal sample from the PWD and AMH pigs that underwent metagenomic analysis

^a^ This sample did not fit the PWD criteria

### Archival samples from DNR and DR cases

Archived faecal samples from four to six-week-old post-weaned pigs, collected from four DNR enteric cases and 11 DR enteric cases (two pigs per case), were also analysed through metagenomics (Tables 2 and 3). The DR cases had previously been diagnosed with enteric disease caused by salmonellosis, enteric colibacillosis and/or rotaviral enteritis. These samples had been archived at -80 °C. Previous viral diagnostic testing initiated on the day of faecal sample receipt included polyacrylamide gel electrophoresis (PAGE) for rotavirus, and PCR for porcine epidemic diarrhoea virus (PEDV) and transmissible gastroenteritis virus (TGEV). PAGE was performed according to standard protocols. The Virotype PEDV/TGEV RT-PCR Kit (Indical Bioscience) was used following the manufacturer’s instructions to test the samples for PEDV and TGEV RNA.

**Table 2:** Information on the four diagnosis-not-reached (DNR) cases investigated in this study

Submission ID	Sample ID	Clinical signs	Other information	Viruses										
				Astro	Sapelo	Entero	Sapo	Kobu	Tescho	Anello	Smaco	Other ssDNA	Parvo	Rota (no of seq. reads)
0077	2625	Wasting and diarrhoea	Chronic necrotic enteritis	+	-	-	+	+	+	+	-	-	+	-
	2629	Wasting and diarrhoea		+	-	-	-	+	-	-	-	-	+	+ (8)
0003	1831	Diarrhoea	Atrophic enteritis on histopathology, some eosinophilic infiltrates	+	+	+	+	+	+	-	+	+	-	-
	1840	Diarrhoea		+	-	+	+	+	+	-	+	+	-	-
0001	2833	Diarrhoea	Atrophic enteritis, colitis, likely viral or possibly coccidial cause, likely secondary bacterial dysbiosis. One case of severe suppurative rhinitis.	+	-	+	+	+	-	-	+	+	-	-
	2822	Diarrhoea		+	-	+	+	+	-	-	+	+	-	-
0041	1087	Diarrhoea and wasting	Diarrhoea (only 1 piglet)	+	-	+	+	+	+	-	-	-	+	-
	1057	Diarrhoea and wasting		+	-	+	-	+	+	-	-	-	-	-

The viruses (viruses genetic materials) identified in each sample using NGS also included in the table

**Table 3:** Information on the eleven diagnosis-reached (DR) cases investigated in this study

Sub. ID	Sample ID	Diagnostic category	Clinical signs	Viruses																
				Astro	Sapelo	Entro	Sapo	Kobu	Tescho	Anello	Picobirna	Smaco	Other ssDNA	Boca	Posa	PPV	Rota (no. of seq reads)	Toro	Adeno	Pasi
0001	7657	Salmonellosis	Diarrhoea	+	+	+	+	+	+	-	-	-	-	+	-	-	+ (110)	-	+	-
	7660	Salmonellosis	Diarrhoea	+	+	+	+	+	-	+	-	+	+	+	-	-	-	-	+	-
0010	5565	Salmonellosis and rotavirus	Malaise, wasting, diarrhoea	+	+	+	+	-	-	-	-	-	+	+	-	-	+ (47)	+	-	-
0017	2723	Enteric colibacillosis and salmonellosis	Malaise and found dead	+	+	+	+	+	-	-	-	+	+	-	-	-	-	-	-	-
	2724	Enteric colibacillosis and salmonellosis	Malaise and found dead	+	+	+	+	+	+	+	-	+	+	-	-	-	-	+	-	-
0020	9719	Salmonellosis and rotavirus	Diarrhoea and wasting	+	+	+	+	+	+	-	-	+	-	-	-	-	+ (4)	-	-	-
	9720	Salmonellosis	Diarrhoea and wasting	+	+	+	+	+	+	+	-	+	-	-	-	-	-	-	-	-
0046	2397	Enteric colibacillosis	Diarrhoea and wasting	+	+	+	+	+	+	+	-	+	-	-	-	+	+ (26)	+	-	-
	2398	Enteric colibacillosis	Diarrhoea and wasting	+	+	+	+	+	+	+	-	+	-	-	-	+	-	-	-	-
0047	0163	Rotavirus, Brachyspira pilosicoli	Diarrhoea, malaise, found dead	+	-	+	-	-	-	-	-	-	-	+	-	-	+ (26)	-	-	-
	0168	Enteric colibacillosis	Diarrhoea, malaise, found dead	+	-	+	-	+	-	-	-	-	-	-	-	-	-	-	-	-
0134	3687	Salmonellosis, colibacillosis, rotavirus	Diarrhoea	+	+	+	+	+	-	-	-	+	-	+	-	-	-	-	-	-
	3738	Salmonellosis, colibacillosis	Diarrhoea	+	+	+	+	+	+	-	-	+	-	+	-	-	-	+	-	+
1527	6344	Rotavirus	Diarrhoea and wasting	+	+	+	+	+	+	+	-	+	-	-	-	-	+ (196)	-	-	-
	6357	Enteric colibacillosis, rotavirus, PRRSV	Diarrhoea and wasting	+	+	+	+	+	+	+	-	+	+	+	-	-	+ (141)	-	+	-
2627	4301	Salmonellosis	Found dead	+	+	+	+	+	+	+	-	-	+	-	-	-	+ (18)	+	-	-
	4302	Enteric colibacillosis and salmonellosis	Found dead	+	+	+	+	+	-	-	-	-	-	-	-	-	+ (8)	+	-	-
0017	7064	Salmonellosis (S Typhimurium and Newport)	Malaise, diarrhoea	+	+	+	-	+	-	-	-	+	+	+	-	+	-	-	-	-
	7068	Salmonellosis (S. Newport) and rotavirus	Malaise, diarrhoea	+	+	+	+	+	-	-	-	+	+	+	-	+	+ (13)	-	-	-
0020	2943	Salmonellosis (S. Typhimurium)	Found dead, diarrhoea	+	+	+	-	+	+	-	-	+	-	-	-	-	-	-	-	-
	2945	Salmonellosis (S. Typhimurium)	Found dead, diarrhoea	+	+	+	-	+	-	-	-	+	-	-	-	-	-	-	-	-

The table also includes diagnostic category, clinical signs and the viruses detected in each sample using NGS. The samples were from diarrhoeic pigs previously diagnosed with enteric disease attributed to salmonellosis, colibacillosis and/or rotavirus infection

### Metagenomic sequencing and analysis

Nucleic acid was extracted from 100 mg of faeces using QIAamp viral RNA mini kit (Qiagen) following the manufacturer’s instructions but omitting the carrier RNA. The extracted nucleic acid, quantified using Nanodrop and Qubit, were submitted to the APHA Central Sequencing Unit for sequencing on an Illumina NextSeq platform. Data analysis was performed using SeqMan NGen, SeqMan and MegAlign software of the DNASTAR Lasergene 17 package (DNASTAR, Inc., Madison, USA). Initial NGS data analysis involved reference-guided assembly using the SeqMan NGen software to screen for viruses. Nucleotide sequences for reference viruses were retrieved from the National Centre for Biotechnology Information (NCBI) genome database, https://www.ncbi.nlm.nih.gov/genome/viruses/, and used as template for this reference-guided assembly. Once viral sequences were identified, the data were re-analysed against entire viral genomes from the corresponding virus family (https://www.ncbi.nlm.nih.gov/Taxonomy/Browser/wwwtax.cgi) to assemble the viral genome where possible and accurately quantify the number of sequences’ reads to the virus. Viral sequence reads were visualised using SeqMan software for validation.

### Statistical analysis

To investigate the association between the percentage of virus sequence reads and clinical status (PWD vs. AMH), we fitted separate linear mixed-effects models for each virus using the *lmer* function from the “lmerTest” R package. Each model included viral measurement as the outcome variable, clinical status as a fixed effect predictor, and a random intercept for submission to account for repeated measures or clustering within submissions. This approach allowed to account for intra-subject variability and correlation structure inherent to the data, which was subsequently quantified by the intra-class correlation coefficient (ICC).

Given the small sample size (36 observations from 9 submissions), we also assessed the statistical power of detecting differences by status for each virus by conducting simulation-based power analyses using the *powerSim* function from the “simr” package. Power simulations were based on the fitted mixed models, using 1000 simulation runs per virus to estimate the probability of detecting a statistically significant effect of clinical status at an alpha level of 0.05. Confidence intervals for power estimates were also calculated. This simulation-based method allowed for realistic power estimation reflecting the complexity of the mixed-effects model and the observed data structure, including the number of observations and variance components.

## Results

### Diagnostic testing for enteric pathogens

Viral laboratory testing carried out for each faecal sample from the PWD and AMH pigs is presented in Table 1. These samples were subjected to metagenomic analysis.

### Virome of PWD and AMH pigs

Sequence reads for the 36 samples received from nine submissions ranged from 11,646,194 to 78,678,762, with an average of 33,311,657 reads per sample (Supplementary Table 1). One AMH sample failed in the Illumina sequencing. Astrovirus, enterovirus, sapelovirus, sapovirus, and kobuvirus had the highest sequence reads of 1,048,770.8, 81,678.1, 119,042.4, 52,416.3 and 19,017.4 respectively. Torovirus and anellovirus had the lowest sequence reads, with an average of only 40.6 and 0.5 reads. We used our well-established NGS data analysis pipeline in this investigation and further refined the methodology while analysing samples from PWD and AMH pigs. This refinement aimed to ensure detection of all known as well as any novel viruses. It was noted that the analysis could be further simplified and its reliability improved by setting the sequence identity threshold for the templated (mapping) analysis to 65%.

Supplementary Table 1: Total sequence reads for each sample and for each of the viruses detected in PWD and AMH cohorts.

A wide spectrum of viruses across at least ten virus families (*Adenoviridae*, *Astroviridae*, *Caliciviridae*, *Parvoviridae*, *Picobirnaviridae*, *Picornaviridae*, *Reoviridae*, *Smacoviridae*,* Anelloviridae* and *Tobaniviridae*) were identified in samples from both PWD and AMH pigs. The detected viruses included astrovirus, enterovirus, kobuvirus, smacovirus, picobirnavirus, sapovirus, parvovirus, posavirus, teschovirus, sapelovirus, rotavirus, anellovirus, torovirus and adenovirus. Co-infection with four viruses, astrovirus, enterovirus, kobuvirus and smacovirus was detected in all samples from PWD and AMH pigs. Additionally, all PWD samples contained sequence reads matching those of sapovirus, parvovirus, and posavirus. Other virus sequences detected in the PWD group, in descending order of frequency, included picobirnavirus (94%), teschovirus (87.5%), sapelovirus (81.25%), rotavirus (75%), adenovirus (37.5%), torovirus (12.5%) and anellovirus (5.5%).

In the AMH group, all samples were also positive for picobirnavirus sequences (in addition to the four viruses mentioned above), compared to 93.75% of PWD samples. Additional virus sequences detected in AMH pigs, in descending order of frequency, included sapovirus and parvovirus (93.75%), teschovirus (87.5%), posavirus (81.25%), rotavirus (62.5%), sapelovirus (56.25%), torovirus (25%), adenovirus (12.5%) and anellovirus (5.7%). Overall, a higher proportion of PWD samples contained sequence reads for sapovirus, parvovirus, posavirus, sapelovirus, rotavirus, adenovirus and torovirus, compared to AMH samples.

No sequence reads matching those of PEDV, TGEV, porcine delta coronavirus (PDCoV), porcine reproductive and respiratory disease virus (PRRSV), porcine circoviruses (PCVs), swine influenza virus (SIV), atypical porcine pestivirus (APPV) or porcine teschovirus-1 (PTV-1) were detected in either PWD or AMH faecal samples.

### Statistical analysis of virus load in PWD and AMH pigs

The percentage of sequence reads for each virus, out of the total number of reads, was calculated as an indication of virus load in each sample (Supplementary Table 2). These percentages were then aggregated by category (PWD or AMH) and virus for analysis. The outcome of the linear mixed models showed low intra-class correlation coefficients (between 0 and 32%) suggesting that the percentage of virus sequence reads are more variable at the individual level within submission than they are between submissions.

Supplementary Table 2: Percentage of sequence reads for each of the viruses detected in the PWD and AMH cohorts.

For several viruses (astrovirus, sapelovirus, enterovirus, sapovirus, adenovirus and possibly sapovirus) a substantially higher viral load was observed in samples from PWD pigs compared to those of AMH pigs (Table 4). However, no statistically significant differences were observed between PWD cases and AMH controls for the percentage of virus sequence reads across all viruses tested (Table 5). The study was limited by the small sample size with low statistical power to detect differences. Simulations showed that the power to detect the observed difference was very low (between 8 and 34%) meaning that these results should be interpreted cautiously, and further studies with larger samples are warranted to confirm these findings.

**Table 4:** Virus load percentages for PWD and AMH pigs

Virus	PWD pigs	AMH pigs
Astrovirus	75.9	14.7
Sapelovirus	6.7	4.1
Enterovirus	7.9	1.9
Sapovirus	2.9	1.9
Kobuvirus	0.7	0.9
Teschovirus	0.1	0.17
Anellovirus	0.00003	0.00002
Picobirnavirus	0.007	0.02
Smacovirus	0.05	0.2
Posavirus	0.3	0.05
Parvovirus	0.29	0.29
Rotavirus	0.01	0.01
Torovirus	0.004	0.0003
Adenovirus	0.01	0.00002

Percentage of each virus sequence reads from that of the total reads were calculated as an indication of the load of each virus in the sample. These percentages were then combined for each category (PWD or AMH)

**Table 5:** Expected difference (β) in the percentage of viruses’ sequence reads between PWD and AMH pigs

Virus	Estimate (β)	95% CI* estimate	*p*-value	ICC^§^ (%)	Power (%)	95% CI* power
Adeno	0.0007	[-0.0003, 0.0017]	0.164	3	32	[29%, 35%]
Astro	3.1414	[-1.7576, 8.0404]	0.201	0	27	[24%, 30%]
Kobu	-0.0116	[-0.0861, 0.0629]	0.751	15	8	[6%, 9%]
PEV	0.2963	[-0.2143, 0.807]	0.244	5	25	[22%, 27%]
PPV	-0.0122	[-0.0482, 0.0237]	0.489	5	11	[9%, 13%]
Picobirna	-0.0009	[-0.0022, 0.0004]	0.176	0	32	[29%, 34%]
Posa	0.0146	[-0.0202, 0.0494]	0.397	2	13	[11%, 15%]
Rota	-0.0004	[-0.002, 0.0013]	0.644	0	9	[7%, 11%]
Sapelo	0.1404	[-0.3505, 0.6313]	0.562	6	9	[8%, 11%]
Sapo	0.0615	[-0.1148, 0.2378]	0.479	23	12	[10%, 14%]
Smaco	-0.0102	[-0.0239, 0.0035]	0.138	0	34	[31%, 37%]
Tescho	-0.0046	[-0.0153, 0.0061]	0.39	0	15	[13%, 18%]
Toro	0.0002	[-0.0001, 0.0004]	0.13	32	32	[30%, 35%]
Total	3.7239	[-1.071, 8.5188]	0.124	0	34	[31%, 37%]

* 95%CI = 95% confidence interval

^§^ ICC = Intra-cluster correlation coefficient

### Virome of archived DNR and DR enteric cases

In the DNR cases, an average of 5.8 viruses were identified in DNR samples, ranging from 4 to 8 viruses in any one sample (Table 2). Co-infection with astrovirus and kobuvirus was detected in all eight samples. Enterovirus and sapovirus sequences were identified in 75% of the samples, teschovirus in 63%, smacovirus and other small ssDNA viruses in 50%, bocavirus in 38% and sapelovirus, anellovirus and rotavirus (only eight sequence reads) in 12.5% of samples. Sequence reads to posavirus, picobirnavirus, torovirus, PEDV, TGEV, PDCoV, PRRSV, PCV, SIV, APPV, PTV-1 and adenovirus were not detected in any of these DNR samples.

In the DR cases, an average of 8.1 viruses were identified in DR samples, ranging from 3 to 12 viruses in any one sample (Table 3). Sequence reads matching those of astrovirus and enterovirus were detected in all samples. Kobuvirus, sapelovirus, sapovirus and smacovirus sequences were also detected in most samples. Teschovirus (not PTV-1) sequences were present in 52% of the samples, while lower levels of sequence reads were identified for bocavirus, parvovirus, rotavirus (4-196 sequence reads), torovirus, adenovirus, pasivirus and other single stranded DNA viruses. Rotavirus sequences were detected in all the five samples that had previously found to be positive for rotavirus RNA by PAGE. Additionally, five other samples that had tested negative by PAGE were found to be positive for rotavirus RNA sequences using NGS. DNA sequences matching those of posavirus, picobirnavirus, PEDV, TGEV, PDCoV, PRRSV, PCV, SIV, APPV or PTV-1 were not detected in any of the samples. Notably, PRRSV was not detected in the faeces of one pig which had previously been diagnosed with active PRRSV infection by PCR on lung tissue (submission ID 1527, sample ID of 6357), as well as enteric colibacillosis and rotavirus infection.

## Discussion

The causes of PWD are multifactorial, typically involving a combination of infectious agents, environmental stressors, immunological and nutritional factors. To analyse the genetic content of microbial communities of PWD cases, we used metagenomic sequencing. It is, however, important to note that this study alone does not conclude a definite association between the presence of a virus and the disease status of the individual pig in which it was detected.

Metagenomic analysis enabled the detection of a broad range of viruses in the faeces of post-weaned pigs. On average, 10.6 viruses were identified in cases of PWD and unaffected AMH pigs. Faeces from PWD cases contained between 10 and 12 viruses, while those from AMH cases contained between 7 and 10 viruses. All the viruses detected are described in the literature and considered common virome components of healthy pigs although some have also been linked to enteric diseases. A similar study investigating the changes in the virome over time in post-weaned pigs with wasting syndrome also identified numerous viruses (*n* = 14) in rectal swabs at various time points [12]. These findings underscore the complex nature of the enteric biome in pigs, disruption of which may be caused by a combination of factors, and that it is not caused solely by the presence of an infectious agent.

Porcine astrovirus (PoAstV) genetic material was found in all samples from PWD, AMH, DNR and DR cases, and accounted for the highest percentage of virus sequence reads. Similarly, in the study by Folgueiras-Gonzalez et al. [12], the majority of enteric viromes in healthy (*n* = 14) and mild to moderately wasting animals (*n* = 8) at seven days post weaning also consisted of astroviruses (62.3% and 67.6%, respectively). PoAstV have a worldwide distribution and are commonly detected and shed by both healthy and diarrheic pigs. Five genetic lineages of PoAstV (PoAstV1–5) have been described [13]. A systematic literature review found a global pooled prevalence of PoAstV infection of 28.19% (95% CI, 21.94%-34.89%), with significant prevalence reported across continents: 26.25% in Asia, 36.19% in Europe and 63.24% in North America [14]. Among the different age groups, nursery pigs (6–10 weeks) had the highest infection rate (63.19%), followed by weaning pigs (3–6 weeks) at 60.00%. PAstV5 has been described in association with enteritis in weaned pigs [15, 16]. Moreover, PAstV4 has been correlated with neonatal diarrhoea and has also been found in high levels in nasal swabs from pigs with unexplained respiratory disease [16, 17]. Several independent studies have identified PAstV3 in the central nervous system of newly weaned pigs with encephalomyelitis and posterior paraplegia [18–20]. Future analysis could explore the genotypes of the PAstVs in these samples.

The other three viruses found in all PWD and AMH cases in this study were enterovirus, kobuvirus and smacovirus. In the study by Folgueiras-Gonzalez et al. [12] other sequences found in healthy animals were enterovirus G (15.9%), porcine sapovirus (12.7%), rotaviruses (8.1%) and, in wasting pigs were porcine sapovirus (13.1), enterovirus G (9.3%), the Circovirus genus (not PCV2/3), rotaviruses (3.8%), and picobirnavirus (3.4%). However, none of the viruses showed a statistically significant difference at seven days post weaning between the two groups of pigs. The ubiquity of asymptomatic enteroviruses, a group of RNA viruses in the *Enterovirus* genus of *Picornaviridae* family, in the global pig population along with its genotypic diversity suggests that enteroviruses may require co-infections and/or additional co-factors to induce clinical manifestations [21]. Folgueiras-González et al., [12] concluded that the presence of enterovirus G and sapovirus three weeks after weaning, when clinical problems peak, suggests that co-infection of the two viruses may contribute to the development of PWD. Kobuviruses, a group of RNA viruses in the *Kobuvirus* genus of *Picornaviridae* family, have been identified in both healthy and diarrheic pigs worldwide, including Europe [22–24], However, their role in diarrhoea is still unknown. Kobuviruses have been identified in co-infection with other viruses such as PCV2, astrovirus, rotavirus A, PEDV, and TGEV [25, 26] complicating interpretation of their potential pathogenic role in diarrhoea. Smacoviruses, previously known as “stool-associated circular viruses”, have recently been classified into the family *Smacoviridae* and include circular ssDNA viruses with genome of 2.3–3.1 kb. They have been identified in faecal samples from both healthy and diarrhoeic animals of various species, including pigs, as well as insect body parts [27]. Smacovirus DNA sequences have been detected in the Archaeal genome by CRISPR analysis, suggesting that smacoviruses may primarily or exclusively infect prokaryotic hosts [28]. Sapoviruses belong to the *Sapovirus* genus within the family *Caliciviridae*. They are non-enveloped viruses with a ssRNA genome. Although a sapovirus strain replicated in the villous epithelial cells and caused intestinal lesions in the proximal small intestines (mainly in duodenal and less in jejunum), causing mild to severe diarrhea, in the orally inoculated neonatal gnotobiotic pigs, the significance of the viruses in different ages of pigs with diarrhea in the field is still undetermined [29].

Picobirnaviruses are bi-segmented dsRNA viruses traditionally considered as opportunistic enteric pathogens of mammals and avian species as they have been detected in faeces of both healthy and diarrhoeic animals [30]. Recent research suggests that *Picobirnaviridae* have bacterial hosts [31] and there is growing evidence that picobirnaviruses are globally highly prevalent and show limited correlation with host species [32].

Kobuvirus, smacovirus and several other viruses, namely parvoviruses (bocavirus and hokovirus), anellovirus and posavirus, identified in this study have not previously been described in pigs in England, although they have been detected in other countries. These viruses are not necessarily new or recent introductions into the pig population in England and may have been endemic but undetected in pigs, given their global presence. Porcine bocavirus (PBoV) and porcine hokovirus (also known as PPV3, partetravirus) are single-stranded DNA viruses in the family *Parvoviridae*. PBoV was first identified in 2009 in Sweden in the lymph nodes of pigs with post-weaning multisystemic wasting syndrome (PMWS) [33]. PBoV has since been reported worldwide in both healthy and diarrhoeic pigs and pigs suffering from respiratory problems and is frequently found in co-infection with other viruses. Its pathogenic role, however, remains unclear. Porcine hokovirus was first reported in 2008 in Hong Kong from samples of different types of tissues collected in slaughter plants with a prevalence of 44.4% [34]. Subsequently, it was detected in Europe, Asia, North America, and South America. Porcine hokovirus was also found in high prevalence (20–54%) in wild pigs from some European countries, likewise, in both young and adult wild pigs. Anelloviruses *are* non-enveloped viruses with circular, negative-sense, single-stranded DNA genomes of 1.7–3.9 kb [35]. The first identified anellovirus, named torque teno virus (TTV), was found in 1997 in the blood sample of a hepatitis patient [36]. Anelloviruses are highly prevalent in diverse mammals, including pigs [37]. So far, they have not been identified as the primary cause of any disease, however, there is some evidence that co-infection with anelloviruses, such as TTV may exacerbate the severity of other viral pathogens. Anelloviruses were the least abundant viruses in the PWD and AMH samples analysed in this study. Porcine stool-associated RNA virus (posavirus) is a single stranded RNA virus, and it is classified as a novel member of the order *Picornavirales*. Posavirus was first identified in the faeces of piglets with diarrhea in the USA in 2011, and it was subsequently detected in the faeces of healthy piglets and adult pigs [38–40].

No viruses were detected in samples from archived DNR and DR cases that were not found in the PWD and AMH pigs, providing no evidence of a new, emerging, or previously unrecognised virus associated with these enteric cases. Detection of multiple viruses in DNR cases underlines the complexity of interpreting the potential role of these infectious agents in such disease syndromes, particularly in the absence of age-matched unaffected controls from pigs on the same and different premises.

Metagenomic analysis also found no evidence of porcine enteric coronaviruses (PEDV, TGEV or PDCoV) providing further evidence that these viruses are not playing a role in enteric disease outbreaks in pigs in England and supporting the findings of other surveillance (APHA, [41]). In addition, non-enteric endemic viruses known to cause disease in pigs, such as PRRSV, PCVs, SIV and APPV were not found in these enteric samples, perhaps unexpectedly for PCV2 although the sample set was relatively small. Apart from one pig with PRRSV, the samples came from pigs which were not suspected to be diseased with any of these pathogens.

Rotavirus was detected by metagenomics in several enteric samples from pigs which had tested negative for rotavirus in the diagnostic PAGE test. These were not necessarily pigs with rotaviral enteritis and suggests that the rotavirus was present below the threshold of detection of the routine diagnostic test. Several of the viruses identified in these enteric samples e.g., astrovirus, sapelovirus, enterovirus and teschovirus, can cause reproductive and/or nervous disease. There are no licensed vaccines in the UK for these pathogens, and they are only occasionally diagnosed as the cause of disease in pigs in England ([42]; APHA [43]). The factors behind expression of disease due to these pathogens are poorly understood, but it is believed that endemic stability exists in most herds, with disease occurring when this endemic immunity is disturbed. The presence of these viruses, some at high frequency, in the faecal samples from diarrhoeic and healthy pigs supports this theory. Exposure of gilts or sows to faeces from young pigs on the same farm is used on some farms to acclimatise gilts to resident pathogens prior to service, and to help in provision of good passive immunity via colostral antibodies, to piglets. Whilst the findings in the study provide evidence for a virological basis to this practice for the viruses mentioned above, it is clear that there are many additional viruses, some of which are poorly understood, present in weaner faeces which may also be transmitted.

Metagenomic analysis identified several viruses with a higher virus load in PWD cases (astro, entero, sapelo, sapo, posa, adeno and toroviruses), but the differences from those of AMH cases were not statistically significant. This may have been due to the limited number of cases in both groups of pigs. It could also be possible that this was due to potential of misclassification of healthy controls. AMH pigs were defined solely by faecal score and clinical appearance on the day of sampling. No information is provided on whether AMH pigs later developed diarrhoea, whether their gut microbiome/virome was already shifting before clinical signs and their immune or nutritional status. As the virome fluctuates rapidly following weaning, some AMH pigs may have been pre-clinical cases, reducing contrast between groups. Also, the inclusion criteria allowed pigs to be sampled after receiving group treatment (e.g., antimicrobials). Though diseased and healthy pigs had to have received the same treatment, treatments such as antibiotics or zinc oxide alternatives may influence viral replication or bacterial co-infections, complicating interpretation. Additionally, this study was a cross-sectional design without longitudinal follow-up, i.e. sampling occurred at a single time point and AMH pigs were not monitored over time. Reliance on percentage of sequence reads as a proxy for viral load may also introduce bias in the analysis as percent read abundance reflects relative rather than absolute viral quantity. Faecal samples differ widely in total nucleic acid composition, microbial biomass, and quality. Not using absolute quantification, or qPCR validation to support metagenomic estimates can weaken the comparisons between pigs and between farms. Therefore, these findings warrant further investigation, such as longitudinal sampling of a larger number of healthy and diseased pigs over time, with as many variables as possible controlled, to elucidate the contribution of the virome and its individual elements to PWD outcomes. A larger number of farms would help provide the statistical power needed to detect differences within datasets. This would enhance understanding of the viral influences and aetiology of postweaning diarrhoea, supporting improved control and prevention strategies.

In summary, this study revealed the complexity of the virus element in the enteric microbiome in post-weaned pigs. Several known porcine viruses were detected for the first time in pigs in England. Significant differences between PWD and AMH cases were not found in the viruses detected and their viral loads. The role of the viruses detected and their interplay with the host and other bacterial or viral flora in inducing PWD, remains unclear. Determining whether co-infections or individual viruses contribute to disease manifestation and/or its severity requires longitudinal field studies and complex statistical analyses, with careful matching of diseased and healthy pigs. Our study supports the potential value of generic diagnostic technologies applied to undiagnosed enteric disease outbreak investigations but also shows the difficulty in interpreting the clinical relevance of viruses detected. Experimental studies could also further explore the clinical and pathological effects of these viruses. Identifying disease-associated virus or viruses may guide the development of targeted vaccines or other interventions for sows or piglets prior to weaning to help prevent post-weaning diarrhoea.

## Supplementary Information

Below is the link to the electronic supplementary material.

Supplementary Material 1.

Supplementary Material 2.

## Data Availability

The NGS datasets generated and analysed during the current study are not publicly available due to the ongoing research project but are available from the corresponding author on reasonable request.

## Abbreviations

AMH: Age-matched healthy
APPV: Atypical porcine pestivirus
DNR: Diagnosis-not-reached
DR: Diagnosis-reached
ICC: Intra-class correlation coefficient
PAGE: Polyacrylamide gel electrophoresis
PBoV: Porcine bocavirus
PCVs: Porcine circovirus
PDCoV: Porcine delta coronavirus
PEDV: Porcine epidemic diarrhoea virus
PoAstV: Porcine astrovirus
PRRSV: Porcine reproductive and respiratory disease virus
PTV-1: Porcine teschovirus-1
PWD: Post weaning diarrhoea
SIV: Swine influenza virus
TGEV: Transmissible gastroenteritis virus
